# Empirical Models of Social Learning in a Large, Evolving Network

**DOI:** 10.1371/journal.pone.0160307

**Published:** 2016-10-04

**Authors:** Ayşe Başar Bener, Bora Çağlayan, Adam Douglas Henry, Paweł Prałat

**Affiliations:** 1 Data Science Laboratory, Ryerson University, Toronto, Ontario, Canada; 2 School of Government and Public Policy, University of Arizona, Tucson, Arizona, United States of America; 3 Department of Mathematics, Ryerson University, Toronto, Ontario, Canada; Universidad Nacional de Mar del Plata, ARGENTINA

## Abstract

This paper advances theories of social learning through an empirical examination of how social networks change over time. Social networks are important for learning because they constrain individuals’ access to information about the behaviors and cognitions of other people. Using data on a large social network of mobile device users over a one-month time period, we test three hypotheses: 1) attraction homophily causes individuals to form ties on the basis of attribute similarity, 2) aversion homophily causes individuals to delete existing ties on the basis of attribute dissimilarity, and 3) social influence causes individuals to adopt the attributes of others they share direct ties with. Statistical models offer varied degrees of support for all three hypotheses and show that these mechanisms are more complex than assumed in prior work. Although homophily is normally thought of as a process of attraction, people also avoid relationships with others who are different. These mechanisms have distinct effects on network structure. While social influence does help explain behavior, people tend to follow global trends more than they follow their friends.

## Introduction

What determines the adoption of beliefs, new technologies, cultural behaviors, and cooperation among human agents? These are all questions of *social learning*, a term that refers broadly to the processes by which a person’s social environment shapes their actions (how a person behaves) and cognitions (how a person thinks). Through social learning, an agent—a term we use to refer to any individual actor within a social network—may adopt the cognitions or behaviors of those they have an opportunity to observe or interact with directly. Combined with forms of individual learning, where an agent learns through their own direct experiences, social learning may provide a more efficient, less costly, and less risky way to learn optimal social behaviors among both human and non-human animals [1–6]. Developing a better understanding of the mechanisms of social learning can thus yield insights into the development and evolution of human culture. At the same time, theories and models of social learning are also of great practical importance as they can also inform how people can more effectively collaborate to solve wrenching social, environmental, and economic problems, such as global climate change [7–11].

Social networks will occupy a prominent role in a comprehensive theory of learning. This is because direct social contacts—who people interact with, talk to, and observe on a regular basis—provide the raw material needed for learning and define a large part of the social environment that constrains what a person could *potentially* learn from other social agents [2, 10, 12–14]. At the same time, products of learning (behaviors and cognitions) also shape the social environments of human agents, since individuals can choose whom they have contact with and potentially select network partners as a function of individual attributes, including behaviors and cognitions [15–19]. In this way, social network structures co-evolve with the products of social learning [20–22]. A better understanding of social learning processes therefore depend on understanding how social agents form networks, are influenced by others within their network, and how these two phenomena work in tandem.

This paper contributes to our understanding of social learning by developing and testing empirical models of how social networks change over time; particularly, how people in a social network choose links on the basis of their own attributes (a phenomenon known as homophily), and how individual attributes are in turn shaped by network structure (a phenomenon known as social influence). Examining these co-evolutionary processes is challenging because it requires data on both networks and attributes that shift over time. While there are many theoretical models of homophily and social influence [23, 24], empirical datasets that allow scholars to test these models and calibrate theoretical expectations based on empirical findings are relatively rare. There are, however, a number of studies that have tested for social influence and homophily within a single social network, such as Kandel’s [25] now-classic study of adolescent friendships, homophily, and socialization in terms of behaviors (drug use and delinquency) and cognitions (political ideology and education goals). Other more recent studies include Lazer’s [26] study of attitudes and communication among federal agency bureaucrats, Aral, Muchnik, & Sundararajan’s [22] study of product adoption and instant messaging among mobile device users, and Lewis, Gonzalez, & Kaufman’s [22] study of tastes and online social networks among college students.

We build on these and other prior studies through the statistical analysis of a large social network of approximately 300,000 mobile device users, for whom longitudinal data are available on both social contacts and individual behaviors over a one-month time period. Individual behaviors in this context are measured as the “tastes” that users exhibit in their use of the Web and mobile applications. In using these data we build on related studies that have used data on tastes and virtual connections (i.e., through online friendships or mobile communications) to examine fundamental social processes occurring in networks [18, 22, 27–29]. By observing how contacts (networks) and tastes (attributes) change over a short time period, we are able to explicitly test hypotheses of network evolution that cannot be examined by looking at cross-sectional data alone.

## Mechanisms of Social Learning: Homophily and Social Influence

**Fig 1:**
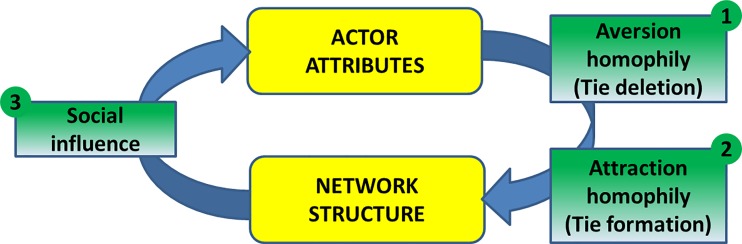
A framework for analysis.

We study the coevolution of network structure and nodal attributes by focusing on three particular social processes as depicted in Fig 1. A distinction here is made between those variables that are broadly thought to interact with one another (variables existing at the framework level—yellow boxes in Fig 1) and the particular mechanisms through which the variables cause change in one another (factors existing at the model level—green boxes in Fig 1). At the framework level, social networks and actor attributes are viewed as having a reciprocal influence on one another. The particular processes at the model level include the formation and deletion of ties based on similarity or differences in agent attributes (homophily), and the diffusion of certain attributes through existing network ties (social influence).

### The role of homophily

Actor attributes may influence the structure of networks through homophily, a widely-studied phenomenon where actors tend to position themselves in networks such that they are connected to others similar to themselves [17, 19]. The formation of ties based on homophily along a wide spectrum of traits is one of the most robust empirical findings in the social networks literature. In social networks, homophily has been observed to operate on socio-economic variables such as race [30, 31], nationality [16], and gender [32, 33], cognitive and behavioral traits such as personal tastes [22], sexual orientation [34], and one’s propensity to cooperate with others [16], as well as physical genetic factors [15]. Homophily has also been studied at the level of social aggregates. For example, it is hypothesized that organizations involved in the policy process tend to coordinate with other organizations sharing their values, interests, and beliefs about policy problems [35, 36]—a hypothesis that is supported by numerous empirical studies [37–40]. Similarly, homophily has been shown to operate at the level of entire cities, such that municipal governments choose other cities as collaborators on the basis of similarity in socio-economic traits as well as shared political ideology [41].

Given the widespread recognition that network structures are determined in part by homophily, then why continue to study this phenomenon? There are at least three answers to this question. First, factors other than homophily help to determine network structure [17, 42], and an analysis of homophily effects must therefore consider how homophily operates in tandem with other structural influences such as optimization [43], seeking out connections with high-degree nodes [44, 45], and the tendency to maintain connections within closed triads, or among “friends of friends” [46–48].

Second, the theoretical mechanisms that lead to homophily in certain contexts are not fully understood. One important distinction in the homophily literature is between homophily that results from a systematic bias for ties with similar individuals (this is known as “choice homophily”) versus homophily that results from the selection of individuals into social contexts based on similar traits (this is known as “structural homophily”). These mechanisms lead to an important puzzle in the study of social networks. Suppose, for instance, that high-school friendship networks exhibit gender homophily in the sense that boys tend to select other boys as friends. This may be because boys simply prefer to befriend other boys (choice homophily). Or it may be because boys have greater opportunity to form friendships because boys tend to engage in the same activities—such as sports teams, which are often gender-segregated (structural homophily). Understanding the influence that both forms of homophily have on network structure is an important theoretical and empirical challenge in networks research [30, 32, 49–51].

Insofar as choice homophily is concerned, it is not understood why people tend to select network partners with similar traits. Kossinets & Watts [51] offer the intuitive explanation that “people form ties with similar others because, rightly or wrongly, they prefer to” (p. 406). Theoretical explanations for this rely on the idea that the creation and maintenance of network ties is costly [43, 52], and these “transaction costs” associated with social ties are reduced when one interacts with others who are similar [53]. Having more in common with one’s social partners makes it easier to form and maintain healthy relationships. Of course, this is also complicated by the fact that different traits might matter differently for homophily, and these differences are likely to be highly dependent on context—so, for example, a person might select their friends based on similarity in political views, but not based on similarity in musical tastes.

Third, homophily is not just a matter of tie formation. Choice homophily in particular may also influence choices about the termination of social ties, or avoidance of certain relationships altogether. In the extensive literature on homophily, scholars almost exclusively consider homophily in terms of the hypothesis *similar individuals tend to form network ties*. This hypothesis captures the literal meaning of homophily, or “love of the same.” On the other hand, there is virtually no work that examines the converse of this hypothesis: *if people are* *not* *similar*, *then they tend to* *not* *form network ties*. The possibility that people have an aversion to those who are dissimilar may work in tandem with “love of the same,” however these are distinct mechanisms that may operate independently of one another [54]. Proof of the former statement—the classic understanding of homophily—does not imply proof of the latter statement. In order to distinguish between these two processes, we use the term “attraction homophily” to refer to the tendency to form ties with those who are similar. We use the term “aversion homophily” to refer to the negation of the homophily process, or avoidance of ties with those who are different.

To illustrate the importance of considering aversion and attraction homophily as distinct social processes, consider Thomas Schelling’s classic work on the emergence of residential segregation [55]. Starting with the observation that many communities exhibit racial segregation (such that people of similar races tend to live spatially proximate to one another) a natural explanation is that people have a strict preference for living in a racially homogenous neighborhood—that is, that individuals exhibit *attraction homophily* in their choice of neighborhood, based on race. On the other hand, Schelling’s model demonstrated that one need not assume that people seek out homogenous neighborhoods for segregation to emerge. Rather, it is sufficient to assume that individuals have a slight bias against being a minority in their local neighborhood—that is, people exhibit a small amount of *aversion homophily* in their choice of residential neighborhood, based on race. When this processes is modeled in a network context it can be shown that slight levels of aversion homophily are sufficient to produce global patterns of network segregation and community structure that are characteristic of many real-world networks [56].

In this paper, we view both types of homophily as independent processes that influence the structure of observed networks: first through the deletion of ties as a result of aversion homophily (Fig 1, process #1), and second, through the formation of new ties as a result of attraction homophily (Fig 1, process #2). As noted above, we study homophily in terms of the various tastes that people have regarding Web browsing behavior and mobile application usage. We therefore consider two homophily hypotheses:

H_1_: In a social network, the likelihood of tie formation between two actors increases with greater similarities in the actors’ attributes.

H_2_: In a social network, the likelihood of tie deletion between two actors increases with greater differences in the actors’ attributes.

### The role of social influence

While actor attributes—such as tastes—shape network structures, networks also have a reciprocal effect on attributes. Many attributes that drive network structures are malleable, particularly cognitive and behavioral attributes such as opinions, values, and cultural traits. Through a process of social influence, the attributes of a particular actor’s network neighbors (those nodes that the actor shares direct connections with) potentially shape the focal actor’s attributes [12, 13, 57]. Social influence is depicted as process #3 in Fig 1.

Studies of social influence are grounded in the observation that a person’s social network partly determines their actions and behaviors. “Social networks” in this sense need not be actual, physical connections, but merely pathways that provide information about other actors’ behavior or cognitions [14]. Scholars have long been interested in developing models of social influence, with early applications in the modeling of how groups with varied opinions on an issue eventually reach a consensus [58]. More recent work seeks to reconcile the idea that the opinions of connected individuals tend to become more similar over time with the observation that we live in a diverse society where people hold diverse opinions. Assuming that individuals always adopt the opinions or other traits of their neighbors always produces consensus, unless networks are fragmented to such an extent that certain groups of people never interact with one another [23, 59]. There are, however, theoretical mechanisms that will allow diversity to emerge even when social influence is at work. For instance, Mäs et al. [59] show that the tendency for people to take on unique, individualistic opinions—but only when the size of a group with a shared opinion become too large—is one possible explanation for the emergence of “opinion clusters” with local consensus but global diversity in opinions.

Applications of social influence models are not limited to investigations of why people hold certain opinions, attitudes, and beliefs, which are the focus of scholars such as [57]. Models of social influence may also be applied to related phenomena such as the adoption of new interests and tastes in an online social network [22], the spread of emotions due to online interactions [60, 61], the adoption of communications technologies [20], sustainability behaviors [62, 63], and even the diffusion of innovations across organizations [64] and governments [65].

In terms of people’s tastes, we hypothesize that social influence processes will cause a convergence in tastes among those connected in a social network, giving our third main hypothesis:

H_3_: In a social network, actors tend to adopt the attributes of others they share direct connections with.

It should be noted that this hypothesis should not apply to all situations where actors exercise—or are prone to—social influence in a network setting. For instance, when individuals learn about scientific information characterized by uncertainty, actors may have differential levels of trust in different information sources due to biased assimilation [66–68], which may create and reinforce polarization in a social network [21].

Another important caveat to this hypothesis is that social influence effects are likely to be mediated by various properties of the network. For instance, social influence (as the degree of convergence in tastes between two actors) is likely to be stronger when actors have higher levels of trust, or more frequent interactions. Actors are therefore more likely to adopt the tastes of others who are more similar to themselves, or with whom they share both direct and indirect ties [46, 69]. Moreover, actors are not only influenced by those in their immediate network neighborhood, but are likely influenced by global shifts as well. These local versus global influences are illustrated by research showing that copycat suicides—that is, suicides resulting from social influence—may be attributed to interpersonal connections as well as news about suicides committed by prominent individuals [70]. In the context of tastes, those in a local network neighborhood likely exert the strongest influences on a particular person’s tastes, however shifts in overall tastes—such as changes in what is “trending” or “in fashion”—are also likely to be an important source of social influence. This possibility is examined below in the analysis of a large empirical dataset on social connections and tastes.

### Data Source

To test the above hypotheses of homophily and social influence in networks, we examine a unique dataset on instant messaging and user tastes from a popular mobile phone platform in the United States. In this analysis, network ties represent contacts between individual mobile phone users—more specifically, if user A has user B in their phone’s instant messaging contact list, then we form an undirected, unweighted network tie from A to B. This definition of links (being in one another’s contact list) and non-links (not being in one another’s contact list) creates a more strict standard for tie formation and deletion than messaging alone, since one must actually add or delete individuals from a contact list in order to form a new tie or delete an existing tie.

Attributes of network nodes are viewed as a combination of Web browser activity and application download history. These behaviors are summarized by a multi-dimensional attribute called “tastes,” which represents the overall interests and behavior of users [18]—at least as they relate to how individuals use their mobile devices. The various dimensions of the taste attribute, called taste categories, were developed by the mobile platform developers and align with the platform’s application categories. While taste categories form a tree structure, in this paper we examine only the seventeen top-level taste categories: lifestyle, finance, business, entertainment, ringtones, photo, news, utilities, health, sports, themes, books, games, social, productivity, education and navigation. The company uses a proprietary algorithm to extract tastes from their users, without any intervention, by mining their activities on the system [71, 72]. This algorithm assigns taste scores between 0 and 1 for individual users over time in each of the seventeen taste categories, where higher values indicate stronger interests in Web searches and applications relevant to the given category. This provides a dynamic view of how the interests and behaviors of users change over time. While the details of the algorithm are private to the mobile device company, the practice of inferring tastes from application and Web use is a widespread practice [71, 72]. These data are typically used to model customer characteristics and provide personalized services, and are particularly useful when there is no opportunity to obtain direct information about mobile device users due to privacy concerns.

It should be noted that tastes are only estimated for users with activity in a given month, and for those users who allow their usage history to be tracked (privacy settings allow users to prevent their usage history from being recorded by the mobile company). Furthermore, this dataset was made available for analysis by the mobile phone company only after it had been completely anonymized. Since these data are not based on direct interactions with mobile device users, and since no individual could be personally identified from the dataset, this project does not fall under the definition of “human subjects” research and was therefore exempt from review by an Institutional Review Board.

The dataset used here is drawn from two snapshots of the contact network and user tastes (attributes) at two points in time: October 19, 2013 and November 22, 2013. A one-month time interval was chosen so that we might observe short-term changes in the network—giving us a view of what might be thought of as the “first derivative” of the longer-term changes in attributes and social networks over time.

Included in this analysis are all mobile platform users in the United States with taste data in a given month—this excludes all users who either had no recorded activity, as well as those users who used their privacy settings to prevent tracking of their usage history. This yields network and attribute data on approximately 300,000 active users, which represents about 10% of all users in the United States between these time periods.

**Fig 2:**
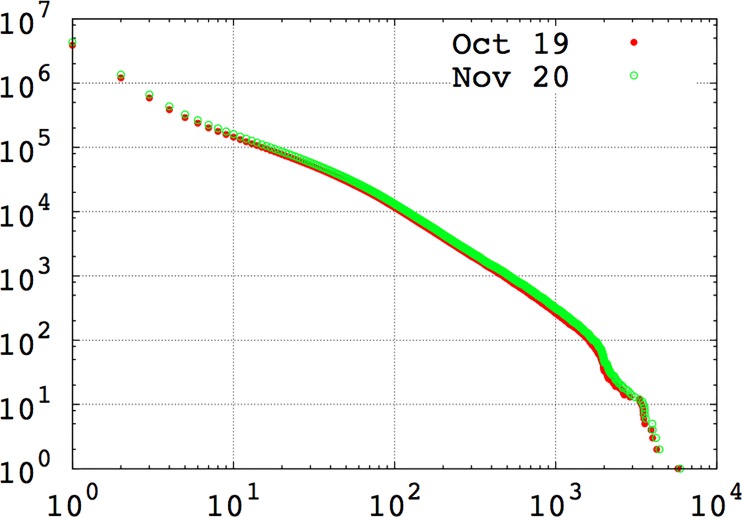
Cumulative degree distributions of two network snapshots.

Table 1 provides descriptive statistics for the two network snapshots, and degree distributions for both networks are depicted in Fig 2. Between the two time periods, there were approximately 426,000 removals of ties and 1.1 million new ties among the active users studied here. If we examine the full network (and not only the users included in this analysis), we see that the network grew in size between the two time periods. Despite this 12% increase in the number of nodes in the network, however, the basic network characteristics remained stable. As seen in Fig 2, both network snapshots are heavy-tailed (with a maximal degree of about six thousand), sparse networks (with an average degree of 3.15), with a large giant connected component (containing 85% of all nodes), and a large number of smaller disconnected components. These characteristics are typical for communication networks among mobile phone users [73, 74].

**Table 1:** Descriptive statistics for two network snapshots.

	October 19^th^	November 22^nd^
Total number of nodes	3.9 million	4.3 million
Total number of edges	6.1 million	6.9 million
Average node degree	3.15	3.15
Size of connected component	3.3 million (85%)	3.7 million (85%)
Total number of components	145,000	172,000

### Logic of hypothesis testing

These data are used to fit statistical models that test our hypotheses about how attribute similarity (or difference) influences tie formation (or deletion), and how tie structures influence changes in tastes. The basic logic underlying these models is to propose a general functional form where the dependent variable (tie formation or deletion in the case of homophily, and taste changes in the case of social influence) is either a logistic or linear function of one or more independent variables. These independent variables include the primary causal effect (i.e., attribute similarity in the case of homophily and sharing a network tie in the case of social influence) and may also include a number of statistical controls—that is, factors that may also influence the dependent variable but are not the primary causal effect under study.

Crucially, the temporal ordering of our observations across two closely-spaced time periods allows us to use these models to infer causal relationships. In particular, homophily hypotheses are tested using functional forms with change in network ties between the first and second time periods as the dependent variable, and with attribute similarities in the first time period as the main independent variable. The social influence hypothesis is testing using a functional form with changes in tastes between the first and second periods as the dependent variable, and tie existence (or absence) in the first time period as the main independent variable.

The functional forms used to test these hypotheses represent assumptions about which variables might potentially influence each dependent variable, however the functions also include parameters (weights on each independent variable) that represent the strength of each causal effect in the model. Models are estimated using standard statistical methods—linear and logistic regression—where parameters are selected that minimize the differences between observed dependent variables and those predicted by the model [75, 76]. The following sections provide more detail on the precise functional forms used to test our three hypotheses.

## Results for Homophily

The structure of these data allow us to explicitly test two distinct views of homophily discussed above—homophily as a process of *attraction* to others with *similar* attributes (H_1_), and homophily as a process of *aversion* from others with *dissimilar* attributes (H_2_). To do this, we take all dyads (i.e., pairs of individual actors) in the network and group them into one of four categories as summarized in Table 2. These groups define whether the dyad contained a newly formed tie (Group 0–1), a deleted tie (Group 1–0), a tie that was present in both time periods (stable ties; Group 1–1), or a tie that was absent in both time periods (stable non-ties; Group 0–0).

**Table 2:** Four categories of dyads.

		*In the second time period…*	
		. . .tie exists	…tie does not exist
***In the first time period*.**	**tie exists**	Stable tie (Group 1–1)	Deleted tie (Group 1–0)
	**tie does not exist**	Newly formed tie (Group 0–1)	Stable non-tie (Group 0–0)

For each dyad in the system, we also calculate two alternative measures of how different each pair of actors are in terms of their attributes. In particular, for network nodes A and B, the *taste difference* between A and B is defined as either the Euclidean distance or the Hamming distance between A and B’s respective tastes (as noted above, each individual taste is a seventeen-dimensional vector). We use a measure of difference rather than similarity because zero has a natural interpretation in a difference measure (i.e., zero means that two nodes have exactly the same attribute). While Hamming distance is estimated based on the number of shared tastes, Euclidean distance is estimated by weighting the shared tastes. Since tastes do change between the two time periods, the *taste difference* variable is recalculated for each snapshot.

Given both the taste difference measures and the grouping of dyads, we are able to test the homophily hypotheses according to the following logic:

1. Under the attraction hypothesis (H_1_): Among all pairs that are *not connected* in the first time period (Groups 0–0 and 0–1), those pairs with greater similarity in tastes are more likely to form a tie between the two periods (that is, belong to Group 0–1).
2. Under the aversion hypothesis (H_2_): Among all pairs that are *connected* in the first time period (Groups 1–1 and 1–0), those pairs with greater dissimilarity in tastes are more likely to delete a tie between the two periods (that is, belong to Group 1–0).

### Homophily as attraction

The attraction hypothesis is tested by fitting a logistic regression model using dyads in Groups 0–0 and 0–1 as the unit of analysis:

$$Y=\frac{1}{1+e^{-(\beta_{0}{+\beta }_{1}\;taste\_diff{+\beta }_{2}\;path\_length)}},\;\text{where}$$ (1)

- *Y* is the probability of tie formation (that is, whether the dyad is a member of Group 0–0 versus Group 0–1),
- β_0_, β_1_, and β_2_ are constant coefficients,
- Variable *taste_diff* is the taste difference between the two nodes in the dyad in the first time period, measured either as Euclidean distance or Hamming distance between taste vectors as described above, and
- Variable *path_length* is the geodesic path length between the two nodes in the dyad in the first time period (when all dyads included in this model are empty). This is included as a control variable, to capture the possible effects of proximity in the social space on tie formation. For example, a large body of research on triadic closure suggests that network actors are more likely to form ties if they both have ties to a common third actor (in which case the value of *path_length* would be two). It is unlikely that the transitivity property will hold uniformly across large, complex networks [77]. However, including this effect in statistical models is useful in that it prevents us from wrongly attributing tie formation decisions to homophily only, when in fact tie formation may be due to a combination of homophily and closeness within the network.

Two important issues with this approach require discussion. First, the use of logistic regression models assume that observations are independent of one another—in other words, the probability of tie formation between one pair of actors is uncorrelated with tie formation between any other two actors in the system. This is not a good assumption, especially given that the same actors appear in multiple dyads. To deal with this problem, we fit models only on a random sample of dyads in the network. Given that this is a relatively small percentage of actor-actor pairs spread out over an entire national market, the chances that we have replicated agents in the sample is very small, and we will be able to safely assume that dyads included in this analysis are, for the most part, independent.

The second issue is that Group 0–0 is extremely large compared to Group 0–1. By comparing samples drawn from both groups we invoke an implicit theory that all disconnected actor pairs have an equal opportunity to form a link between the two time periods. This is, of course, unrealistic. Thus, we are in need of a way to narrow the population of stable non-tie dyads (Group 0–0) to a subgroup of dyads that we can realistically assume had an opportunity to form a link between the two time steps.

**Fig 3:**
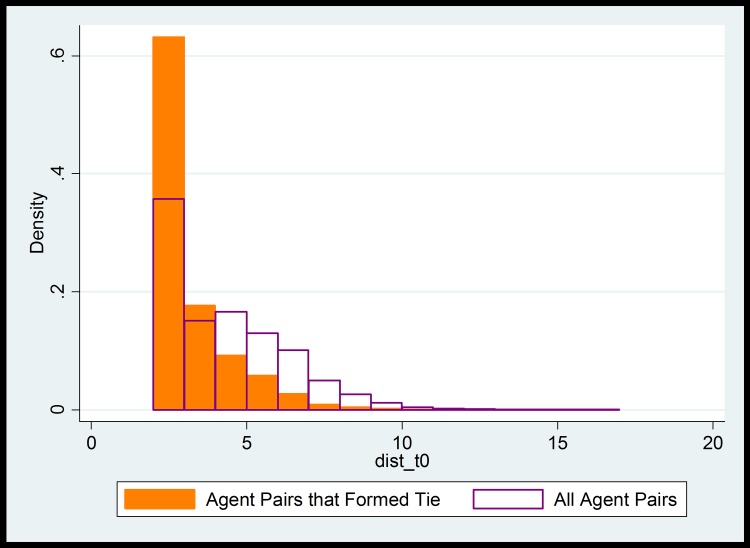
Distributions of geodesic path lengths in first time period. Orange region represents the density of geodesic path lengths between disconnected actors who formed a tie before the second time period (members of Group 0–1). White region represents the geodesic path lengths between disconnected actors that did not form a tie before the second time period (members of Group 0–0).

Our approach is to look at the geodesic path distances between actor pairs in Group 0–0 in the first time step, and compare them with the geodesic path distances between actor pairs in Group 0–1 (newly formed ties). These distributions, depicted in Fig 3, suggest that there is a maximum path length beyond which it can be safely assumed that actors are in completely different communities, with no opportunity to form ties with one another.

Examining differences between these distributions enables us to develop a heuristic, based on path length, for whether two actors have an opportunity to form a tie. The 98^th^ percentile of path lengths among actors with newly-formed ties (Group 0–1) is six, meaning that fewer than two percent of all actors who formed a tie started with greater than six degrees of separation. This suggests that dyads excluding from Group 0–0 all dyads where actors are separated by more than 6 degrees of separation narrows the universe of Group 0–0 dyads to those who plausibly had an opportunity to form a tie.

Table 3 summarizes the results obtained from fitting the logistic regression model (Eq 1) after the exclusion of Group 0–0 dyads with path length seven or greater, and a random sampling of 20% of all remaining dyads from each group. As predicted (H_1_), we find strong negative coefficients on both concepts of taste differences. This indicates that as taste differences in the first time period increase, the probability of tie formation between the two time periods decreases. This effect is strongly significant (p < 0.001 in both models).

**Table 3:** The effect of attribute differences on tie formation and tie deletion.

	*Model 1 (DV = tie formation)*	*Model 2 (DV = tie formation)*	*Model 3 (DV = tie deletion)*	*Model 4 (DV = tie deletion)*
Euclidean distance	-0.135		0.176	
	(0.041)		(0.032)	
Hamming distance		-0.053		0.072
		(0.016)		(0.013)
Path length in first time period	-1.056	-1.056		
	(0.035)	(0.035)		
Constant	4.048	3.998	-0.873	-0.820
	(0.164)	(0.156)	(0.085)	(0.078)
N	3,540	3,540	3,328	3,328
pseudo-R^2^	0.2767	0.2766	0.0070	0.0068

Standard errors reported in parenthesis.

### Homophily as aversion from those who are different

Testing for the aversion aspect of homophily is more straight-forward, since it is not necessary to estimate the population of ties that are candidates for deletion. Dyads in Groups 1–0 and 1–1 all have an existing tie on the first time period, all of which are treated as candidates for deletion. Similar to the analysis reported above, the probability of tie deletion is estimated as a logistic function of attribute differences in the first time period, using dyads drawn from Groups 1–0 and 1–1 as the unit of analysis:

$$Z=\frac{1}{1+e^{-(\beta_{0}{+\beta }_{1}\;taste\_diff)}},\;\text{where}$$ (2)

- *Z* is the probability of tie deletion (that is, whether the dyad is a member of Group 1–0 versus Group 1–1),
- β_0_ and β_1_ are constant coefficients, and
- Variable *taste_diff* is the taste difference as described above.

This logistic regression model (2) is estimated using another random sample of 20% of all dyads to deal with the potential problem of interdependence among observations. Results from this logistic regression analysis are reported as Models 3 and 4 in Table 3.

**Fig 4:**
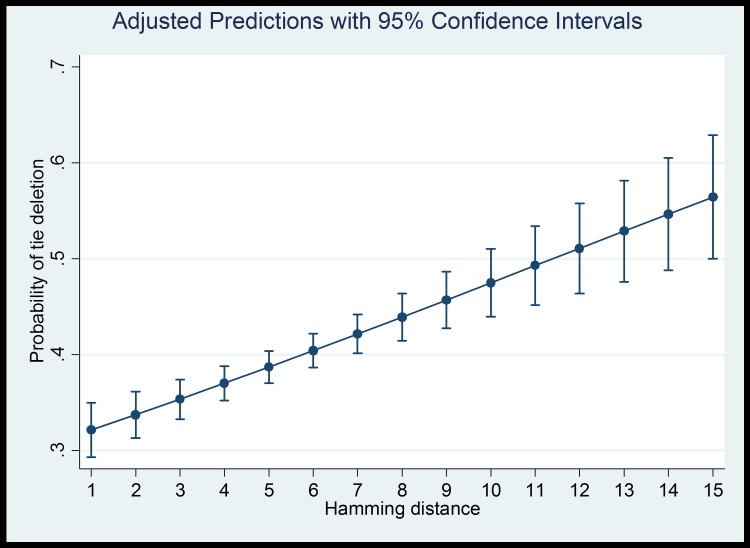
Effect of attribute differences on the predicted probability of tie deletion.

As with H_1_, these results lend strong support for the aversion hypothesis (H_2_). Note that the effect of both taste difference measures—Euclidean distance and Hamming distance—are both positive and significantly different from zero (p<0.001 in both models). This indicates that, as distances increase, the probability of tie deletion also increases. This effect is also represented graphically in Fig 4. The models including Hamming distance allow for a slightly easier interpretation, since coefficients in these models tell us how dissimilarity in each additional taste dimension will change the probability of tie formation or deletion.

## Results for Social Influence

The longitudinal nature of this dataset also allows us to directly test the social influence hypothesis (H_3_), that individuals tend to adopt the attributes of those they are connected to in a social network. In the context of this study, we examine whether changes in tastes between the two time periods can be explained by social contacts in the first time period.

Our approach is to view shifts in each user’s tastes between the two time periods as a function of two factors: 1) shifts in the average tastes of the individual that the user is connected to (referred to as the user’s *local center of mass*), and 2) global shifts in the average tastes of all users (referred to as the system’s *global center of mass*). More formally, for a single taste element T, a particular user’s change in tastes is modeled as follows:

$$T_{2}-T_{1}=a(L_{2}(T)-L_{1}(T))+b(G_{2}(T)-G_{1}(T))+e,\;\text{where}$$ (3)

- *T*_*1*_ and *T*_*2*_ are the user’s tastes during the first and second time periods,
- *L*_*1*_*(T)* and *L*_*2*_*(T)* are the user’s local centers of mass at the first and second time periods respectively for taste element *T* (that is, the average taste *T* of individuals the user is connected to in a given time period),
- *G*_*1*_*(T)* and *G*_*2*_*(T)* are the global centers of mass for taste *T* in the first and second time periods, respectively,
- *a* and *b* are constant coefficients ranging from 0 to 1, inclusive, and
- *e* is an error term capturing motivations for change not accounted for in the other terms.

According to this model, we assume that a user’s shift in tastes will follow some constant fraction of the shift in the global center of mass (that is, users will “follow” global trends to some degree), and at the same time users will also follow trends within their local network neighborhood. The constant coefficients *a* and *b* are weights that indicate the degree to which a user is influenced by these global and local trends, where a zero value of *a* or *b* indicates that users are not at all influenced by shifts in local or global tastes, respectively, and a value of one indicates that users are strongly influenced by shifts in these tastes. Finally, the error term, *e*, captures a user’s individual propensities for changing tastes; that is, factors other than one’s social environment as measured by the *L* and *G* variables.

Since the model above (3) expresses changes in user tastes between the two time periods as a linear combination of observed local and global shifts in tastes, the coefficients *a* and *b* may be estimated using a standard ordinary least squares (OLS) regression. Table 4 reports the results of seventeen regression models, one for each of the measured user tastes. While the goodness of fit varies greatly across tastes, the estimated weights on local and global shifts in tastes (*a* and *b*, respectively) are significantly different from zero in all models (p < 0.001). This suggests that the tastes of users are indeed influenced by their social environment.

**Table 4:** Estimated influences of global and local centers of mass on user tastes.

*Model*	*Effect of change in global*	*Effect of change in local*	*R*^*2*^
*(taste element)*	*center of mass (b)*	*center of mass (a)*	
lifestyle	0.223	0.768	0.58
	(0.012)	(0.002)	
finance	0.897	0.077	0.06
	(0.044)	(0.001)	
business	0.870	0.070	0.05
	(0.037)	(0.001)	
entertainment	0.869	0.079	0.06
	(0.032)	(0.001)	
ringtones	0.955	0.019	0.02
	(0.258)	(0.000)	
photo	0.860	0.085	0.06
	(0.031)	(0.001)	
news	0.908	0.051	0.04
	(0.041)	(0.001)	
utilities	0.792	0.154	0.12
	(0.023)	(0.001)	
health	0.904	0.073	0.06
	(0.046)	(0.001)	
sports	0.903	0.050	0.04
	(0.051)	(0.001)	
themes	0.976	0.017	0.01
	(0.068)	(0.000)	
books	0.890	0.060	0.04
	(0.061)	(0.001)	
games	0.908	0.060	0.05
	(0.032)	(0.001)	
social	0.445	0.545	0.43
	(0.014)	(0.002)	
productivity	0.664	0.287	0.22
	(0.022)	(0.001)	
education	0.877	0.073	0.05
	(0.041)	(0.001)	
navigation	0.785	0.159	0.12
	(0.026)	(0.001)	

Standard errors are reported in parenthesis.

**Fig 5:**
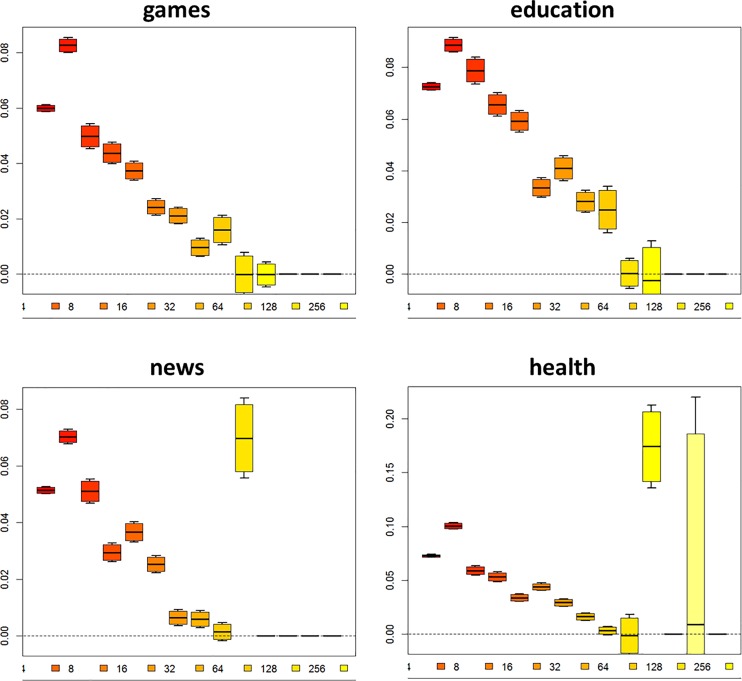
Distribution of changes in user tastes as a function of number of social contacts. Boxplots represent distributions of individual user shifts in tastes between October 19^th^, 2013 and November 22^nd^, 2013. Distributions are conditional on degree of user on October 19^th^. Boxplots exclude outliers.

As noted above, other aspects of the network may exercise an influence over these processes of social influence; that is, as a function of their local network, some users may be more or less susceptible to social influences. One such influence is the degree of nodes—it turns out that users with more contacts also tend to experience smaller shifts in their own tastes. This pattern is depicted graphically in Fig 5, which shows for a given taste the distribution of users’ change in tastes between the two time periods (vertical axis) against the degree of the user in the first time period (horizontal axis). Shifts in four tastes are shown here for illustrative purposes; however, this decreasing trend is seen across all measured tastes. This suggests that as individuals are exposed to greater numbers of social contacts, they tend to be less influenced by their social environment.

## Conclusion

This paper contributes to our understanding of social learning and network evolution through the analysis of a large mobile contact network, coupled with data on the tastes of individuals, over two points in time. The unique structure of these data allow for an examination of how tastes influence the formation of new contacts (attraction homophily, H_1_) and the deletion of contacts (aversion homophily, H_2_), and how contacts in turn influence the tastes of actors in the network (social influence, H_3_).

We find evidence in support of all three hypotheses. In terms of attraction homophily, the overall similarity between actors in terms of their tastes is a significant predictor that they will form a tie, controlling for the degree of separation between these actors in the network. Similarly, aversion homophily exists in that, among connected actors, those that terminate ties tend to be more different than actors who do not cut their ties. These results make an important contribution to the literature on homophily. On the one hand, the result that “love of the same” is a basis for network formation is consistent with many other studies of homophily in social networks—here we demonstrate that this phenomenon applies to the addition of new mobile device contacts in a large, complex network. On the other hand, the question of whether the converse of homophily—avoidance of those who are different—is virtually unstudied in the literature on social networks. We show that this is a significant effect, even though the amount of variation in tie deletion explained by divergent tastes is small relative to the amount of variation in tie formation explained by shared tastes (compare, for instance, the pseudo-R^2^ values of 27.67% and 0.70% in Table 3, Models 1 and 3, respectively). This may be explained by the idea that tie formation is easy compared to tie deletion, and other factors will need to come into play before “aversion homophily” has the chance to influence network structure. Overall, these results suggest that future work on network evolution should focus on models of tie deletion as well as tie formation—if a particular effect leads actors to form ties, one should not assume the opposite effect is at work.

There is also strong evidence that tastes diffuse through existing links—that is, that shifting individual tastes do tend to follow changes in the average tastes of one’s network contacts. Interestingly, shifts in global trends appear to explain far more of the variation in individual tastes than shifts in the tastes of one’s direct network contacts. This finding is contrary to expectation for network scholars who argue that people are more strongly influenced by those they are socially close to. It seems that trends within local neighborhoods do matter—that is, social influence does shape tastes—however these data also suggest that people follow trends more than they follow their friends. This is a useful contribution to the literature because it suggests network scholars should consider how global contextual factors influence behavioral changes in tandem with local, personal network ties. If linked actors tend to independently follow global shifts and models do not take these trends into account, then we may attribute more importance than we should to changing tastes within a given actor’s local network neighborhood. In other words, we may overestimate the effect of social influence on behavioral change.

This paper builds on a large and vibrant literature on network evolution. This research shows that it is useful for scholars—both in theoretical models as well as in empirical work—to make a distinction between the rationales for tie formation and tie deletion. While in this dataset we find similar strengths of taste similarity on the formation and deletion of ties, in other contexts homophily may not operate in both directions. It may be possible that actors will exhibit a small preference for homophily in the creation of new ties networks, but the maintenance of ties with dissimilar alters may be very costly.

We also show that social influence is a complex phenomenon that is unlikely to reduce to a simple process of averaging across the tastes (or other attributes) of one’s network neighbors. The effect of social influence differs greatly depending on the attribute in question. In the case of tastes, some attributes exhibit relatively low levels of social influence while others are quite prone to change as a result of local and global shifts. Social influence is also mediated by other factors such as degree; those who have relatively few connections tend to be, on average, much more strongly influenced by their local network neighborhood.

Finally, this research illustrates the potential of working with large datasets on contacts and mobile device usage. The strength of the homophily and influence effects seen in this research suggest that we are, indeed, examining a dataset that lend insights into other types of social networks—for instance, networks involving actual physical interactions between people, who vary their worldviews and behaviors based on those they interact with socially.

At the same time, it is important in future work to explicitly examine the degree to which datasets such as these are appropriate laboratories to test hypotheses of social learning and network evolution. This dataset allows for an examination of how the tastes of mobile device users influence the tastes of any particular user. However, this is probably just one part of the true social environment that explains tastes. Similarly, in the future it will be important to examine a more broad and fine-grained set of attributes that both determine and are influenced by network structure. As defined here, tastes are one useful measure of human behavior, but future work should aim to extend this to attribute variables that help us to further understand salient environmental, economic, and political problems—such as understandings of scientific information, political ideology, or norms of cooperative behavior. Overall, in this research we contribute to theories of social learning by showing how learning is at least partly conditional on social network structures, which both constrain and enable the exposure of individual agents to new information, technologies, and behaviors. As we show in this paper, an essential part of a theory of learning will be robust models of how network structures co-evolve with learned attributes, with a particular emphasis on the roles of homophily—both aversion and attraction homophily—and social influence.
